# Supplementary material for: Mindfulness, Interoception, and Olfaction: A Network Approach
Source: Brain Sci. 2020 Nov 29;10(12):921. doi: 10.3390/brainsci10120921 (PMC7760383; doi:10.3390/brainsci10120921)
Supplement: Supplementary file 1 [file brainsci-10-00921-s001.pdf]

Supplemental materials

Questionnaires (FMI, SPANE, MAIA, subjective acuity)

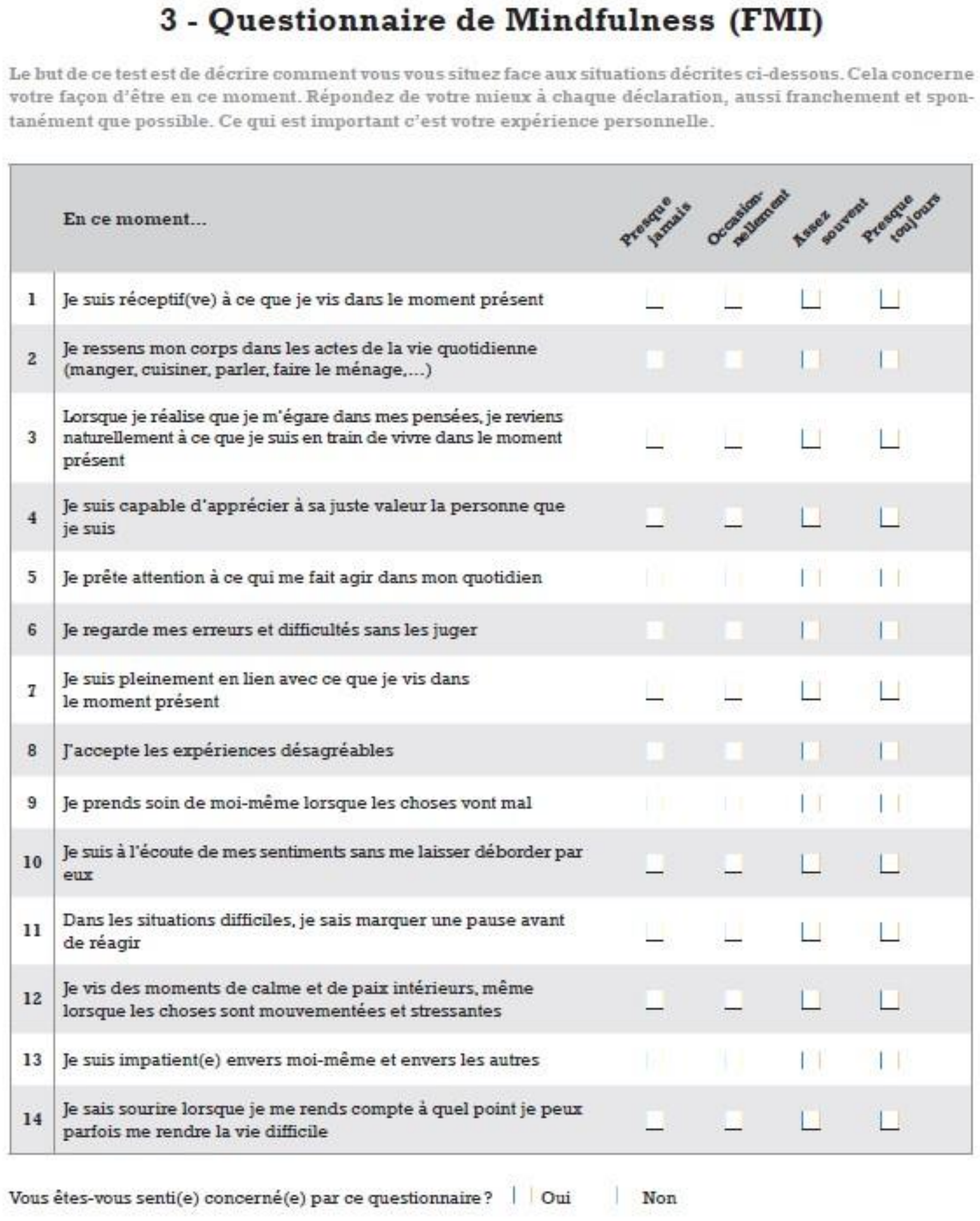

3 - Questionnaire de Mindfulness (FMI)

Le but de ce test est de décrire comment vous vous situez face aux situations décrites ci-dessous. Cela concerne votre façon d'être en ce moment. Répondez de votre mieux à chaque déclaration, aussi franchement et spontanément que possible. Ce qui est important c'est votre expérience personnelle.

	En ce moment...	Presque jamais	Occasion-nellement	Assez souvent	Presque toujours
1	Je suis réceptif(ve) à ce que je vis dans le moment présent	☐	☐	☐	☐
2	Je ressens mon corps dans les actes de la vie quotidienne (manger, cuisiner, parler, faire le ménage,...)	☐	☐	☐	☐
3	Lorsque je réalise que je m'égare dans mes pensées, je reviens naturellement à ce que je suis en train de vivre dans le moment présent	☐	☐	☐	☐
4	Je suis capable d'apprécier à sa juste valeur la personne que je suis	☐	☐	☐	☐
5	Je prête attention à ce qui me fait agir dans mon quotidien	☐	☐	☐	☐
6	Je regarde mes erreurs et difficultés sans les juger	☐	☐	☐	☐
7	Je suis pleinement en lien avec ce que je vis dans le moment présent	☐	☐	☐	☐
8	J'accepte les expériences désagréables	☐	☐	☐	☐
9	Je prends soin de moi-même lorsque les choses vont mal	☐	☐	☐	☐
10	Je suis à l'écoute de mes sentiments sans me laisser déborder par eux	☐	☐	☐	☐
11	Dans les situations difficiles, je sais marquer une pause avant de réagir	☐	☐	☐	☐
12	Je vis des moments de calme et de paix intérieurs, même lorsque les choses sont mouvementées et stressantes	☐	☐	☐	☐
13	Je suis impatient(e) envers moi-même et envers les autres	☐	☐	☐	☐
14	Je sais sourire lorsque je me rends compte à quel point je peux parfois me rendre la vie difficile	☐	☐	☐	☐

Vous êtes-vous senti(e) concerné(e) par ce questionnaire ? ☐ Oui ☐ Non

Figure S1. FMI

5 - Evaluation de la conscience de soi

Vous trouverez ci-dessous une liste de propositions. Merci de bien vouloir indiquer la fréquence à laquelle chacune de ces propositions s'applique à vous dans votre vie de tous les jours.

		Jamais				Toujours
1	Lorsque je suis tendu(e), je perçois où la tension se situe dans mon corps.	☐	☐	☐	☐	☐
2	Lorsque je me sens mal dans mon corps, je le remarque.	☐	☐	☐	☐	☐
3	J'identifie/je remarque à quel endroit de mon corps je me sens confortable	☐	☐	☐	☐	☐
4	Je perçois les changements dans ma respiration, par exemple lorsqu'elle ralentit ou accélère.	☐	☐	☐	☐	☐
5	Je ne perçois pas (j'ignore) les tensions physiques ou l'inconfort jusqu'à ce qu'ils ne deviennent sévères.	☐	☐	☐	☐	☐
6	Je me détache des sensations d'inconfort.	☐	☐	☐	☐	☐
7	Lorsque je ressens de la douleur ou de l'inconfort, je m'efforce de les surmonter.	☐	☐	☐	☐	☐
8	Lorsque je ressens une douleur physique, cela me stresse	☐	☐	☐	☐	☐
9	Je commence à me soucier que quelque chose n'aille pas dès que je ressens le moindre inconfort.	☐	☐	☐	☐	☐
10	Je peux percevoir une sensation corporelle déplaisante sans m'en inquiéter.	☐	☐	☐	☐	☐
11	Je peux prêter attention à ma respiration sans être distrait(e) par les choses qui arrivent autour de moi.	☐	☐	☐	☐	☐
12	Je peux rester conscient de mes sensations corporelles intérieures même lorsqu'il se passe beaucoup de choses autour de moi).	☐	☐	☐	☐	☐
13	Lorsque je suis en conversation avec quelqu'un, je peux porter attention à ma posture.	☐	☐	☐	☐	☐
14	Je peux rediriger mon attention sur mon corps si je suis distrait(e).	☐	☐	☐	☐	☐
15	Je peux détourner mon attention de mes pensées pour la tourner vers mon corps (vers mes sensations corporelles).	☐	☐	☐	☐	☐
16	Je peux conserver la conscience de l'ensemble de mon corps même lorsqu'une partie de moi-même éprouve de la douleur ou de l'inconfort	☐	☐	☐	☐	☐
17	Je suis capable de focaliser mes pensées de façon consciente sur mon corps dans son entier.	☐	☐	☐	☐	☐
18	Je perçois comment mon corps change lorsque je suis en colère.	☐	☐	☐	☐	☐
19	Lorsque quelque chose ne va pas dans ma vie, je peux le ressentir dans mon corps.	☐	☐	☐	☐	☐
20	Je remarque que mes sensations corporelles changent après une expérience apaisante.	☐	☐	☐	☐	☐
21	Je perçois que ma respiration devient dégagée et aisée lorsque je me sens confortable.	☐	☐	☐	☐	☐
22	Je perçois comment mon corps change lorsque je me sens heureux(se)/joyeux(se).	☐	☐	☐	☐	☐
23	Lorsque je me sens débordé(e), je peux trouver un endroit calme à l'intérieur de moi.	☐	☐	☐	☐	☐
24	Lorsque je prends conscience de mon corps, je ressens une sensation de calme.	☐	☐	☐	☐	☐
25	Je peux utiliser ma respiration pour réduire la tension.	☐	☐	☐	☐	☐
26	Lorsque je suis pris(e) dans mes pensées, je peux calmer mon esprit en me concentrant sur mon corps /ma respiration.	☐	☐	☐	☐	☐
27	Je suis à l'écoute de mon corps concernant mon état émotionnel.	☐	☐	☐	☐	☐
28	Lorsque je suis stressé(e), je prends le temps d'explorer comment mon corps se sent.	☐	☐	☐	☐	☐
29	J'écoute mon corps afin de m'informer sur ce que je dois faire.	☐	☐	☐	☐	☐
30	Je suis chez moi dans mon corps.	☐	☐	☐	☐	☐
31	Je sens que mon corps est un endroit sûr.	☐	☐	☐	☐	☐
32	Je fais confiance à mes sensations corporelles.	☐	☐	☐	☐	☐

Figure S2. MAIA

2 - Expériences positives et négatives (SPANE)

Les énoncés suivants représentent ce que vous pourriez ressentir dans votre vie de tous les jours. Pour chaque sensation, cochez la case qui correspond le mieux à ce que vous pensez. Utilisez le dernier mois comme période pour considérer chaque situation.

	Dans quelle mesure avez-vous expérimenté les sensations suivantes ?	Très rarement	Rarement	Parfois	Souvent	Très souvent /Toujours
1	Sensations positives					
2	Sensations négatives					
3	Je me suis senti bien					
4	Je me suis senti(e) mal					
5	Des choses agréables					
6	Des choses désagréables					
7	Je me suis senti(e) heureux(se)					
8	Je me suis senti(e) triste					
9	J'ai ressenti de la peur					
10	Je me suis senti(e) joyeux(se)					
11	Je me suis senti(e) en colère					
12	J'ai ressenti de la satisfaction					

Figure S3. SPANE

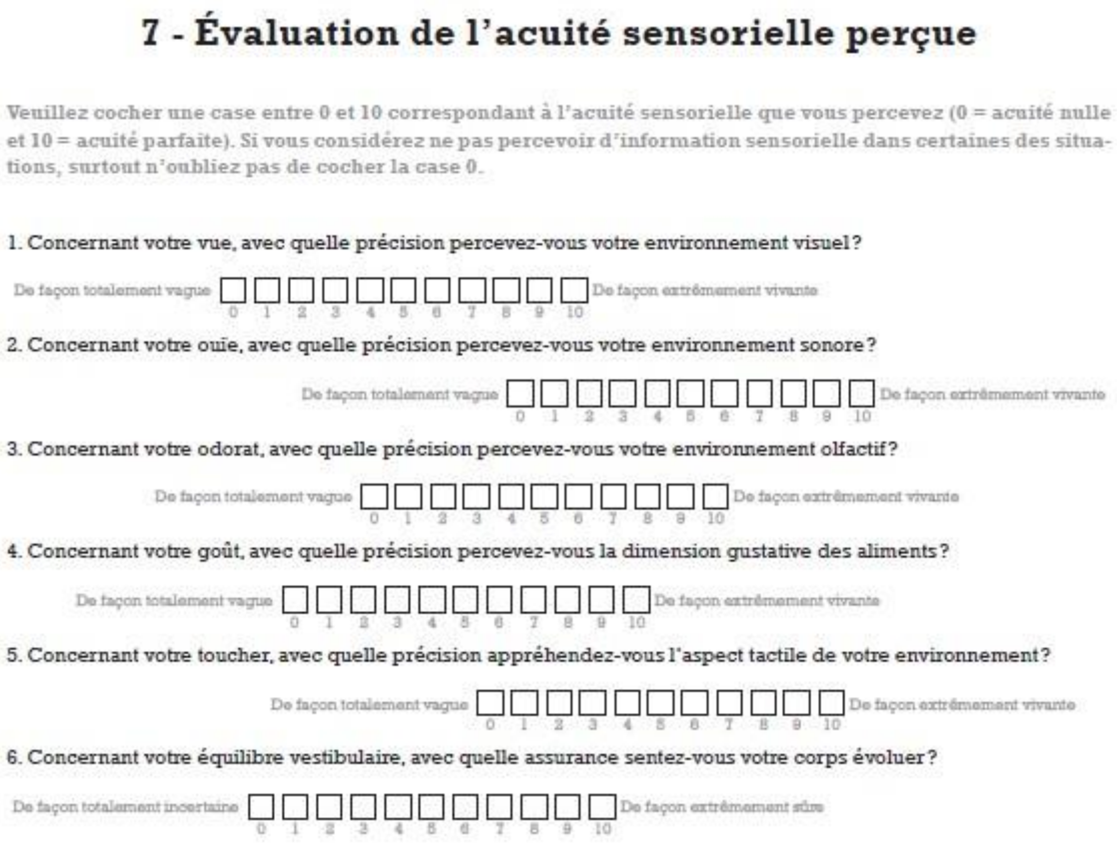

7 - Évaluation de l'acuité sensorielle perçue

Veuillez cocher une case entre 0 et 10 correspondant à l'acuité sensorielle que vous percevez (0 = acuité nulle et 10 = acuité parfaite). Si vous considérez ne pas percevoir d'information sensorielle dans certaines des situations, surtout n'oubliez pas de cocher la case 0.

1. Concernant votre vue, avec quelle précision percevez-vous votre environnement visuel ?

De façon totalement vague ☐ ☐ ☐ ☐ ☐ ☐ ☐ ☐ ☐ ☐ ☐ De façon extrêmement vivante
0 1 2 3 4 5 6 7 8 9 10

2. Concernant votre ouïe, avec quelle précision percevez-vous votre environnement sonore ?

De façon totalement vague ☐ ☐ ☐ ☐ ☐ ☐ ☐ ☐ ☐ ☐ ☐ De façon extrêmement vivante
0 1 2 3 4 5 6 7 8 9 10

3. Concernant votre odorat, avec quelle précision percevez-vous votre environnement olfactif ?

De façon totalement vague ☐ ☐ ☐ ☐ ☐ ☐ ☐ ☐ ☐ ☐ ☐ De façon extrêmement vivante
0 1 2 3 4 5 6 7 8 9 10

4. Concernant votre goût, avec quelle précision percevez-vous la dimension gustative des aliments ?

De façon totalement vague ☐ ☐ ☐ ☐ ☐ ☐ ☐ ☐ ☐ ☐ ☐ De façon extrêmement vivante
0 1 2 3 4 5 6 7 8 9 10

5. Concernant votre toucher, avec quelle précision appréhendez-vous l'aspect tactile de votre environnement ?

De façon totalement vague ☐ ☐ ☐ ☐ ☐ ☐ ☐ ☐ ☐ ☐ ☐ De façon extrêmement vivante
0 1 2 3 4 5 6 7 8 9 10

6. Concernant votre équilibre vestibulaire, avec quelle assurance sentez-vous votre corps évoluer ?

De façon totalement incertaine ☐ ☐ ☐ ☐ ☐ ☐ ☐ ☐ ☐ ☐ ☐ De façon extrêmement sûre
0 1 2 3 4 5 6 7 8 9 10

Figure S4. Subjective acuity

Causal networks

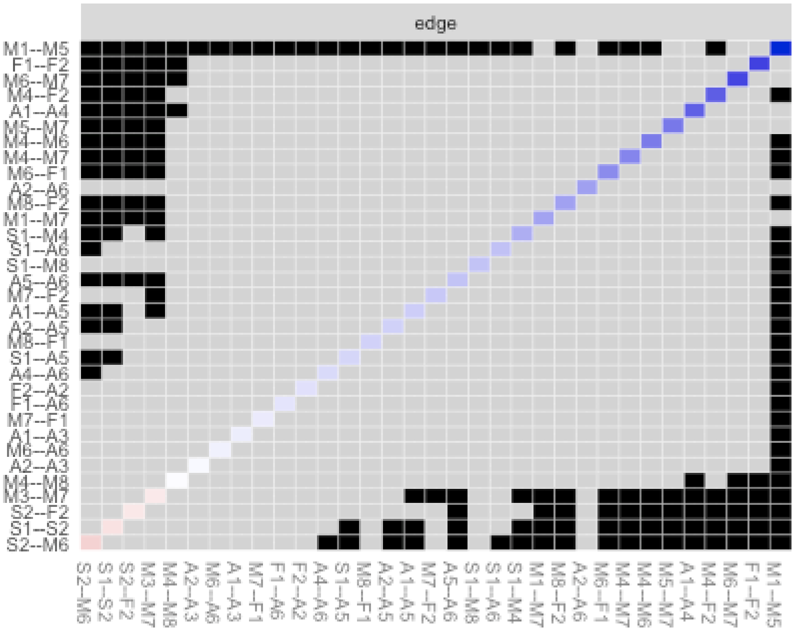

Figure S5. Bootstrapped difference test ($\alpha = 0.05$) according to all variables of all participants between the weight of non-zero connections in the causal network. Grey boxes indicate connections that do not differ significantly from each other. Black boxes indicate significant interactions.

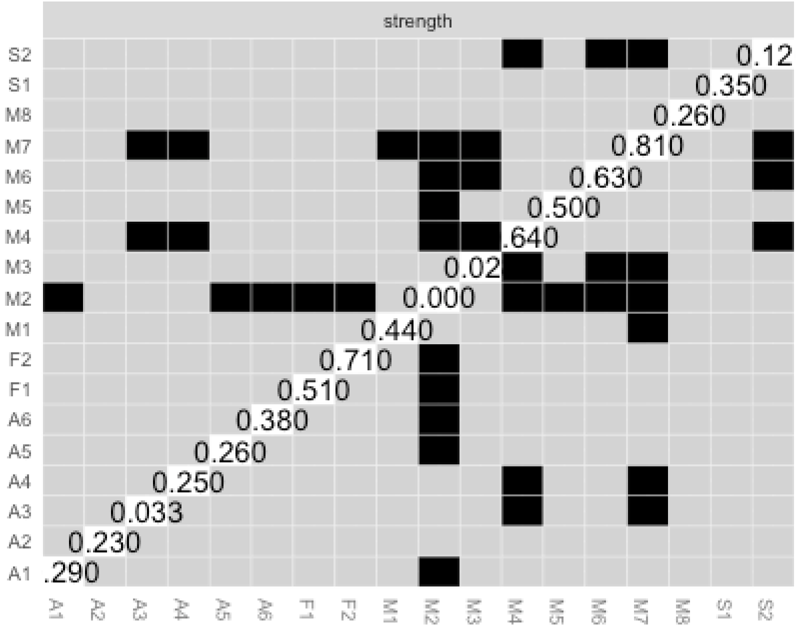

Figure S6. Boostrapping difference test ($\alpha = 0.05$) according to all the variables of all participants between non-zero node forces in the causal network according to the high MD group. Grey boxes indicate connections that do not differ significantly from each other. The higher the number, the more central the corresponding node is. Black boxes indicate significant interactions.

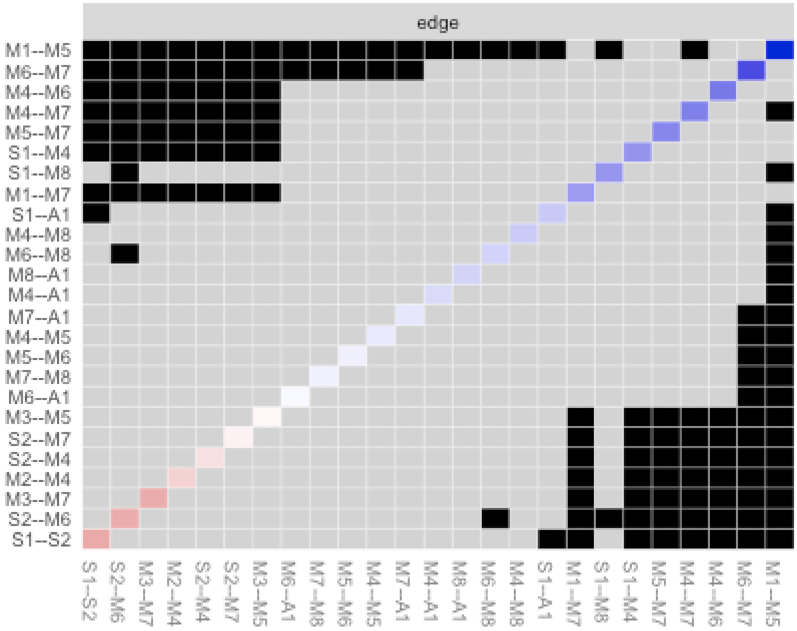

Figure S7. Bootstrapped difference test ($\alpha = 0.05$) according to all participants sample between the weight of non-zero connections in the causal network. Grey boxes indicate connections that do not differ significantly from each other. Significant interactions are reported in black boxes.

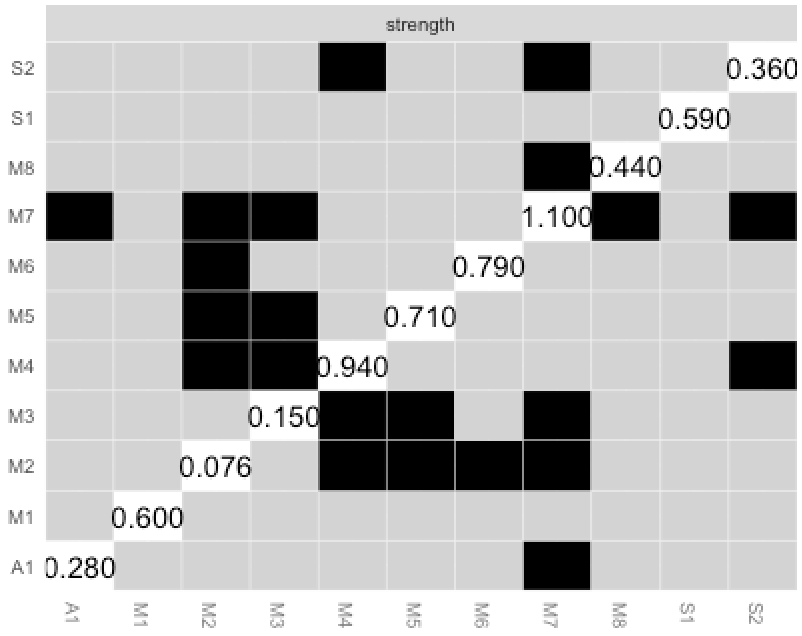

Figure S8. Boostrapping difference test (α = 0.05) according to all participants sample between non-zero node forces in the causal network according to the high MD group. Grey boxes indicate connections that do not differ significantly from each other. The higher the number, the more central the corresponding node is. Black boxes indicate significant interactions.

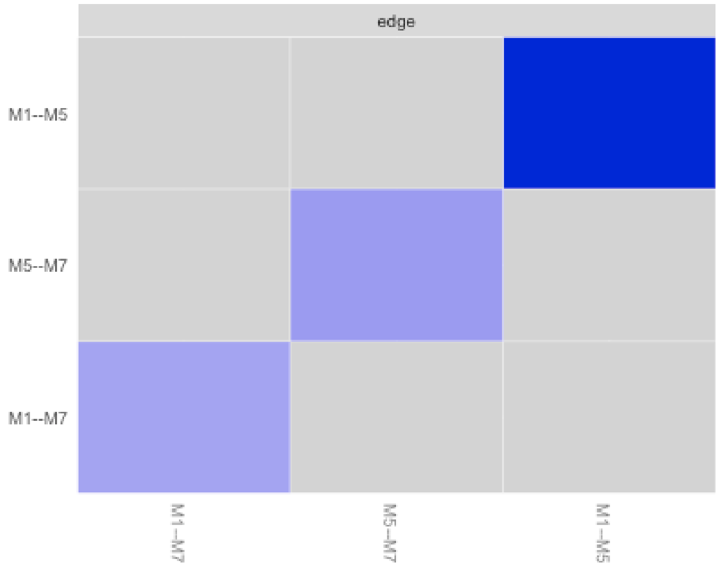

Figure S9. Bootstrapped difference test (α = 0.05) according to the low MD group between the weight of non-zero connections in the causal network. Grey boxes indicate connections that do not differ significantly from each other. No significant interaction is reported.

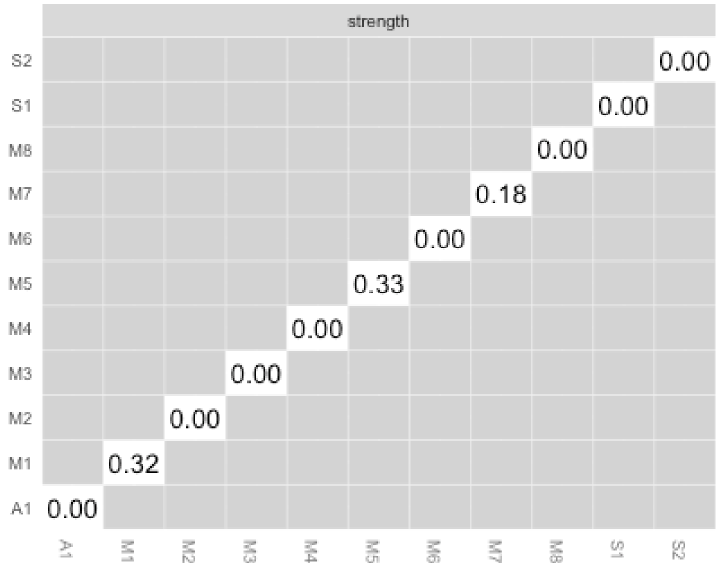

Figure S10. Boostrapping difference test (α = 0.05) between non-zero node forces in the causal network according to the low MD group. Grey boxes indicate connections that do not differ significantly from each other. The higher the number, the more central the corresponding node is. No significant interaction is reported.

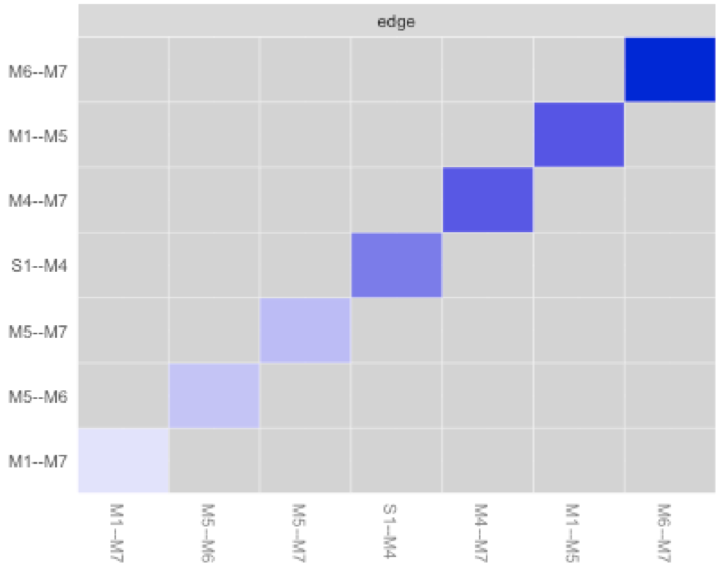

Figure S11. Bootstrapped difference test (α = 0.05) according to the high MD group between the weight of non-zero connections in the causal network. Grey boxes indicate connections that do not differ significantly from each other. No significant interaction is reported.

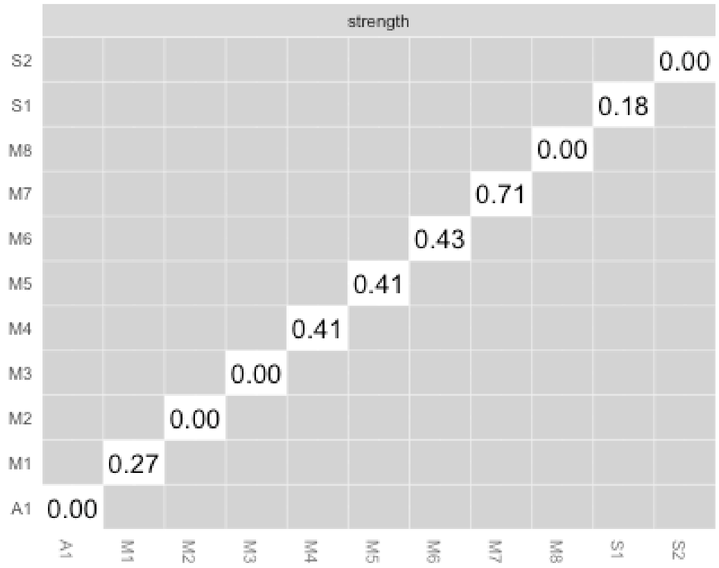

Figure S12. Boostrapping difference test (α = 0.05) between non-zero node forces in the causal network according to the high MD group. Grey boxes indicate connections that do not differ significantly from each other. The higher the number, the more central the corresponding node is. No significant interaction is reported.

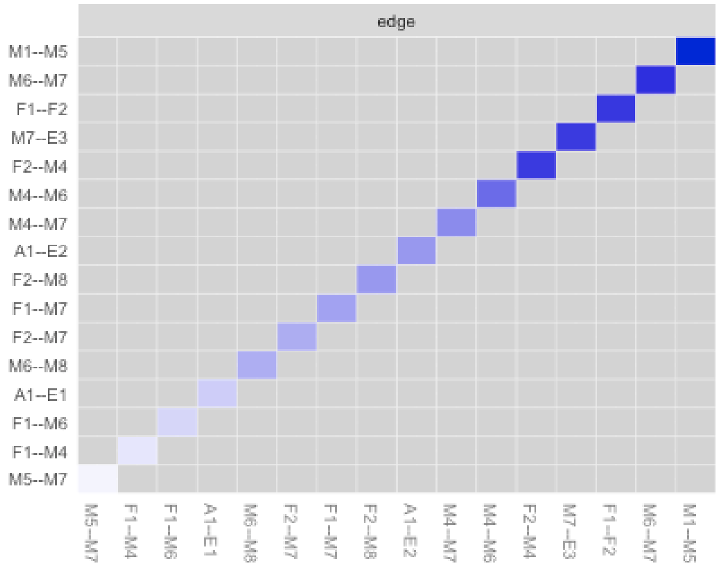

Figure S13. Bootstrapped difference test (α = 0.05) according to sub-sample who completed the ETOC between the weight of non-zero connections in the causal network. Grey boxes indicate connections that do not differ significantly from each other. No significant interaction is reported.

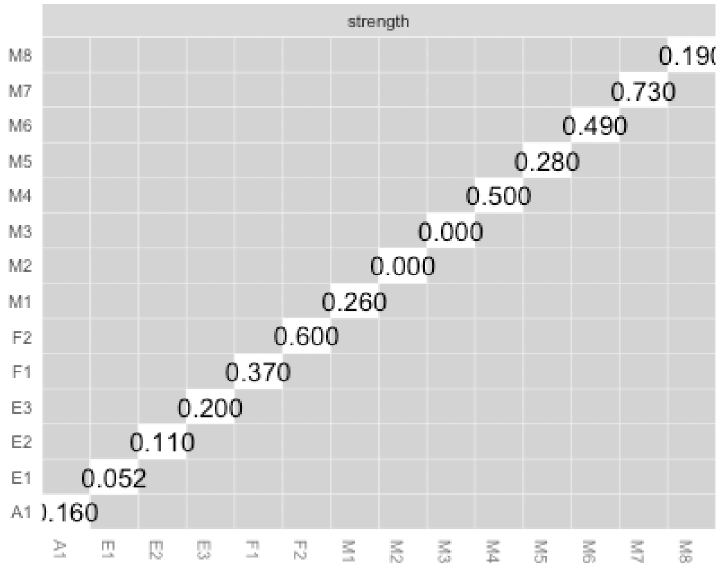

Figure S14. Boostrapping difference test (α = 0.05) according to sub-sample who completed the ETOC between non-zero node forces in the causal network according to the high MD group. Grey boxes indicate connections that do not differ significantly from each other. The higher the number, the more central the corresponding node is. No significant interaction is reported.
